# PRSS55 plays an important role in the structural differentiation and energy metabolism of sperm and is required for male fertility in mice

**DOI:** 10.1111/jcmm.16116

**Published:** 2021-01-08

**Authors:** Feng Zhu, Wen Li, Xinli Zhou, Xu Chen, Meimei Zheng, Yiqiang Cui, Xiaofei Liu, Xuejiang Guo, Hui Zhu

**Affiliations:** ^1^ State Key Laboratory of Reproductive Medicine Department of Histology and Embryology Nanjing Medical University Nanjing China; ^2^ Department of Pathology The First People's Hospital of Changzhou Changzhou China

**Keywords:** energy metabolism, male infertility, PRSS55, sperm motility, spermiogenesis

## Abstract

Orderly and stage‐specifically expressed proteins are essential for spermatogenesis, and proteases play a key role in protein activation and function. The present study aimed to investigate serine protease 55 (PRSS55), which was reported to play a role in sperm‐uterotubal junction (UTJ) migration and sperm‐zona pellucida (ZP) binding. We found that PRSS55 was specifically expressed in testicular spermatids and epididymal spermatozoa. By constructing knockout mice targeting all transcripts of *Prss55*, we demonstrated that deletion of *Prss55* resulted in a serious decline of male fertility, with significantly increased sperm malformation and decreased sperm motility. In *Prss55* ^−/−^ mice, increased structural abnormality, including deficient “9 + 2” microtubules, damaged peripheral dense fibre, and defective mitochondrial cristae, were found in sperm. In addition, sperm showed decreased expression of electron transfer chain molecules and lower ATP contents. These could be the potential causes of the astheno/teratozoospermia phenotype of the *Prss55* ^−/−^ mice, and provided new evidence for the previously reported impaired sperm‐UTJ migration. Moreover, preliminary studies allowed us to speculate that PRSS55 might function by activating type II muscle myosin in the testis, which is involved in many processes requiring motivation and cytoskeleton translocation. Thus, PRSS55 is essential for the structural differentiation and energy metabolism of sperm, and might be a potential pathogenic factor in astheno/teratozoospermia. Our results provide an additional explanation for the male sterility of *Prss55* ^−/−^ mice, and further reveal the role of PRSS55.

## 1 INTRODUCTION

Infertility is commonly encountered in clinical practice, which not only affects marriages and families, but is also a worldwide reproductive health problem. Approximately 20% of couples of childbearing age worldwide suffer infertility, among which male factors account for about 50%.^1^ Spermatozoa are the ultimate executor of the male reproductive process. The study of spermatogenesis has been a hot topic and focus in the field of reproductive biology, and understanding the pathogeneses of spermatogenesis disorders plays an important role in improving the diagnosis and treatment of male infertility.

Studies have shown that spermatogenesis is regulated by intricate gene and protein networks, and orderly and stage‐specifically expressed genes and proteins play a key role.^2^ Proteases are of interest, especially the largest serine protease family. They usually activate target substrate proteins by hydrolysing specific peptide segments, and then affect other downstream proteins, thus executing a cascade regulatory role.^3^ Some members of this family play a cooperative role with other proteins.^4^ Thus, proteases may participate in complex protein networks. A series of studies have shown the important roles of serine proteases in various physiological functions.^5^

According to the Human Protein Atlas database analysis and literature review, male reproductive organs contain the most abundant serine proteases (Table S1), which suggests the importance of serine proteases in male reproduction. Some studies have focused on the potential roles of serine proteases in testicular spermatogenesis. For example, serine proteases PRSS41‐43 were found to be involved in spermatocyte meiosis, and inhibition of PRSS41‐43 induced meiosis arrest and germ cell apoptosis.^6^, ^7^ In addition, some PRSS members play an important role in sperm function. For example, deficiency of PRSS37 resulted in defective sperm migration into the oviduct and reduced the in vitro sperm‐egg interaction, resulting in infertile male mice.^8^, ^9^ Mice lacking PRSS21 were subfertile; their spermatozoa exhibited decreased motility and an impaired ability to bind the zona pellucida (ZP) of oocytes.^10^, ^11^, ^12^ However, some PRSS members, such as PRSS44, PRSS46, PRSS51 and PRSS54, were reported to be dispensable for male fertility; their deletion had no effect on mouse fertility and the routine testing indices of testis and sperm.^13^, ^14^ However, more members of the PRSS family need to be studied to reveal their role in male reproduction.

The present study focused on PRSS55, whose role in male reproduction is a matter of debate. Khan et al found that *Prss55* KO mice were fertile and thus concluded that PRSS55 is dispensable for male fertility.^15^ However, Shang et al and Kobayashi et al proposed that PRSS55 is essential for male reproduction; they found that deficiency of PRSS55 resulted in defective sperm migration from the uterus into the oviduct.^14^, ^16^ However, there were inconsistencies in their results. For example, Shang et al showed that *Prss55* ^−/−^ sperm presented defective recognition and binding to zona‐intact or zona‐free oocytes, but the in vitro fertilization rate was not affected. However, Kobayashi et al only found defective binding of *Prss55* ^−/−^ sperm to zona‐intact oocytes, and the in vitro fertilization rate was decreased significantly. In terms of knockout strategy, all the three research teams deleted two transcripts of *Prss55* in the testes; however, the phenotypes were not exactly the same.

In the present study, we designed a single guide RNA (sgRNA) targeting exon 3 of *Prss55*, which could achieve knockout in mice by targeting all four transcripts of *Prss55*. We then conducted a series of experiments using this KO mouse model, aiming to determine the necessity of PRSS55 in male fertility and to explore whether there were more abnormal phenotypes, thus providing a more comprehensive analysis of PRSS55's functions.

## 2 MATERIALS AND METHODS

### 2.1 Animals

Two pairs of primers (Primer1, Primer2) were designed to verify all possible *Prss55* transcripts in the testes. The primer sequences are listed in Table S2. *Prss55* knockout (KO) mice were generated via Cas9/RNA‐mediated gene targeting, as previously described,^17^ and we designed an sgRNA for zygote injection that could target the knockout mice for all transcripts of *Prss55*. All mice were housed in a specific pathogen‐free animal facility with a light:dark cycle of 12:12. All the animal experiments in this study were approved by the Institutional Animal Care and Use Committees of Nanjing Medical University, Nanjing, China.

### 2.2 Assessment of fertility

To determine the fertility of the mice, a male was mated with two females. The female mice were separated from the male after the formation of a normal vaginal plug was detected. The number of offspring produced per female was recorded. The mean values for each genotype combination were then plotted.

### 2.3 Sperm parameter analysis

Sperm were extracted and incubated in human tubal fluid (HTF) medium (Easycheck, Nanjing, China, M1120) supplemented with 10% foetal bovine serum (FBS) at 37°C. A 10‐μL aliquot of the sperm samples was evenly distributed on a glass chamber slide and analysed using a Computer Assisted Sperm Analyzer (CASA) via the IVOS II system (Hamilton Thorne, Beverly, MA, USA).

### 2.4 Measurement of sperm ATP in vitro

Sperm samples were washed twice, resuspended in lysis buffer, vortexed, and then placed on ice. ATP was measured using luminometric methods via commercially available luciferin/luciferase reagents according to the manufacturer's instructions (ATP Assay Kit; Beyotime Biotechnology, Shanghai China) in a luminometer (TD‐20/20; Turner Designs, San Jose, CA, USA). An average of 3 × 10^7^ sperm were used for ATP analysis.

### 2.5 Histological analysis

The testes and epididymis tissues from adult wild‐type (WT) and *Prss55* ^−/−^ mice were fixed in modified Davidson's Fluid and embedded in paraffin. Sections were cut at 5‐μm thickness and then stained with haematoxylin and eosin (H&E). The coloured sections were digitized under an Axioscope microscope (Carl Zeiss AG) equipped with a motorized X‐Y sensitive stage. Spermatogenic cells in six spermatogenic tubules per sample of stages VII–VIII and XI were counted. For sperm morphology analysis, sperm was washed three times in phosphate‐buffered saline (PBS), mounted on slides, and stained with haematoxylin and eosin (H&E). At least 200 sperm cells were analysed per sample.

### 2.6 Electron microscopy

Sperm samples from adult WT and *Prss55* ^−/−^ mice were fixed in 0.1 M phosphate buffer (pH 7.3) containing 2.5% glutaraldehyde overnight. Sample preparation and processing, comprising post‐fixing 4% (vol/vol) glutaraldehyde‐fixed sperm with 2% (wt/vol) OsO4 and then embedding them in Araldite, were performed by the Electron Microscopy Laboratory of Nanjing Medical University. Transmission electron microscopy imaging was conducted on a JEM‐1010 transmission electron microscope (JEOL, USA, Inc.).

### 2.7 RT‐PCR and qRT‐PCR analyses

Total RNA was extracted from mouse tissues using Trizol (Invitrogen), following the manufacturer's directions, and mRNA was reverse transcribed to cDNA using a Prime Script™ RT Master kit (Takara). PCR (polymerase chain reaction) was performed using 2 × Taq Plus Master Mix (Vazyme, Nanjing, China, P212‐01), with primers specific for mouse *Prss55*. Quantitative real‐time PCR (qPCR) was performed using a SYBR Premix Ex Taq II Kit (Takara), with primers specific for mouse *Mtatp* (encoding mitochondrially encoded ATP synthase), *Mtcyb* (mitochondrially encoded cytochrome B), *Cox2* (cytochrome C oxidase 2), *Cox3* (cytochrome C oxidase w), *Adam3* (a disintegrin and metallopeptidase domain 3), *Myl3* (myosin, light polypeptide 3), *Myh9* (myosin, heavy polypeptide 9), *Myh10* (myosin, heavy polypeptide 10), *Myl6* (myosin, light polypeptide 6)and Actb (β‐actin). Primer sequences are listed in Table S2.

### 2.8 Western blotting analysis

Proteins were extracted using RIPA Lysis Buffer (Beyotime Biotechnology) and quantified using a Pierce BCA Protein Assay Kit (Thermo Scientific). After denaturation at 100°C for 5 minutes, the samples were subjected to 10% sodium dodecyl sulphate‐polyacrylamide gel electrophoresis (SDS‐PAGE) and transferred to Immobilon PVDF membranes (Millipore, Billerica, MA, USA). The membranes were subsequently blocked with 5% nonfat dry milk powder in TBST (20 mM Tris‐HCl pH 7.5, 150 mM NaCl, 0.1% Tween20). Primary antibodies were applied overnight at 4°C, followed by incubation with secondary antibodies for 1 hour at room temperature. Immunoreactive protein bands were detected using an ECL kit (Thermo Scientific, Waltham, MA, USA). All antibodies used are shown below: Rabbit polyclonal anti‐PRSS55 (Abcam; Cat. No: 121215; 1:500), anti‐Flag (EnoGene, E12‐026‐4; 1:1000), anti‐BCL2 apoptosis regulator (BCL2) (Proteintech; Cat. No: 12789‐1‐AP; 1:1000), anti‐BCL2 associated X, apoptosis regulator (BAX) (Proteintech, Cat. No: 50599‐2‐Ig; 1:1000), anti‐caspase 3 (Proteintech, Cat. No: 19677‐1‐AP; 1:1000), anti‐outer dense fibre of sperm tails 1 (ODF1) (Abcam, Cat. No: 204577; 1:500), mouse monoclonal anti‐β‐actin (Merck Millipore; Cat. No: MAB1501; 1:8000), horseradish peroxidase (HRP)‐conjugated goat anti‐rabbit IgG (Thermo Fisher Scientific; Cat. No: 31460; 1:1500) and HRP‐conjugated goat anti‐mouse IgG (Thermo Fisher Scientific; Cat. No: 31430; 1:3000).

### 2.9 Immunofluorescence analysis

Sections were obtained from samples that were fixed using Davidson's Fluid and embedded in paraffin. The sections were blocked in 1% bovine serum albumin and then incubated overnight at 4°C with Rabbit polyclonal anti‐PRSS55 (Abcam, Cat. No: 121215; 1:50). The sections were then incubated with secondary antibodies comprising AlexaFluor 488 anti‐rabbit (Abcam, Cat. No: 150077, 1:500) for 2 hours at room temperature, following previously published protocols.^18^ Slides were viewed under an LSM700 confocal microscope (Carl Zeiss AG).

### 2.10 Apoptosis detection analysis

Apoptosis detection of spermatogenic tubules and cells was achieved using a terminal deoxynulceotidyl transferase nick‐end‐labelling (TUNEL) BrightRed Apoptosis Detection Kit (Vazyme), following the manufacturer's directions. Slides were viewed under an LSM700 confocal microscope (Carl Zeiss AG).

### 2.11 Immunoprecipitation from cell culture extracts

HEK293T cells were transfected with plasmids encoding a flag‐tagged PRSS55 fusion protein (for PRSS55 overexpression) using Lipofectomine 3000 (Thermo Fisher Scientific). Two days after transfection, cells were lysed using Pierce IP Lysis Buffer (Thermo Fisher Scientific) supplemented with 1% (vol/vol) protease inhibitor mixture (Selleck，Houston，TX, USA) for 45 minutes at 4°C, and then clarified using centrifugation at 12 000 *g* for 30 minutes. The lysates were precleared with 50 μL of Protein A/G magnetic beads (Selleck) for 1 hour at 4°C. The precleared lysates were incubated overnight with anti‐Flag antibodies (EnoGene, E12‐026‐4; 2 μg) at 4°C. Lysates were then incubated with 100 μL of Protein A/G magnetic beads for 3 hours at 4°C. The beads were washed three times with immunoprecipitation (IP) Lysis Buffer and eluted with Pierce IgG Elution Buffer (Thermo Fisher Scientific). Samples were boiled in sodium dodecyl sulphate (SDS) loading buffer before being subjected to SDS‐PAGE.

### 2.12 Mass spectrometry analysis

For mass spectrometry analysis, elution was followed by in‐solution digestion. Briefly, elution was first achieved using ultrafiltration through a 10‐kDa filter membrane in protein extraction buffer (8 mol/L urea, 75 mmol/L NaCl, 50 mmol/L Tris, pH 8.2, 1% [vol/vol] EDTA‐free protease inhibitor, 1 mmol/L NaF, 1 mmol/L β‐glycerophosphate, 1 mmol/L sodium orthovanadate, 10 mmol/L sodium pyrophosphate). Cysteine residues were reduced using dithiothreitol (DTT) at a 5 mmol/L final concentration for 25 minutes at 56°C, followed by alkylation in 14 mmol/L iodoacetamide for 30 minutes at room temperature in the dark. Unreacted iodoacetamide was quenched with DTT for 15 minutes. Lysates were then diluted down to 1.6 mol/L urea with 25 mmol/L Tris, pH 8.2. CaCl_2_ was added to a final concentration of 1 mmol/L and the samples were digested overnight at 37°C with trypsin at a concentration of 5 ng/μL. Trifluoroacetic acid at a final concentration of 0.4% was added to stop the digestion. The peptides were desalted using OASIS HLB Extraction Cartridges (Waters) before mass spectrometric analysis.

### 2.13 Statistical analysis

All assays were performed at least three times. Statistical analyses were evaluated using SPSS19.0 (IBM Corp., Armonk, NY, USA). The statistical significance of differences in the mean values was assessed using the *t* test. Values of *P* < .05 were considered statistically significant.

## 3 RESULTS

### 3.1 Expression and location of PRSS55 in adult mice

**Figure 1:**
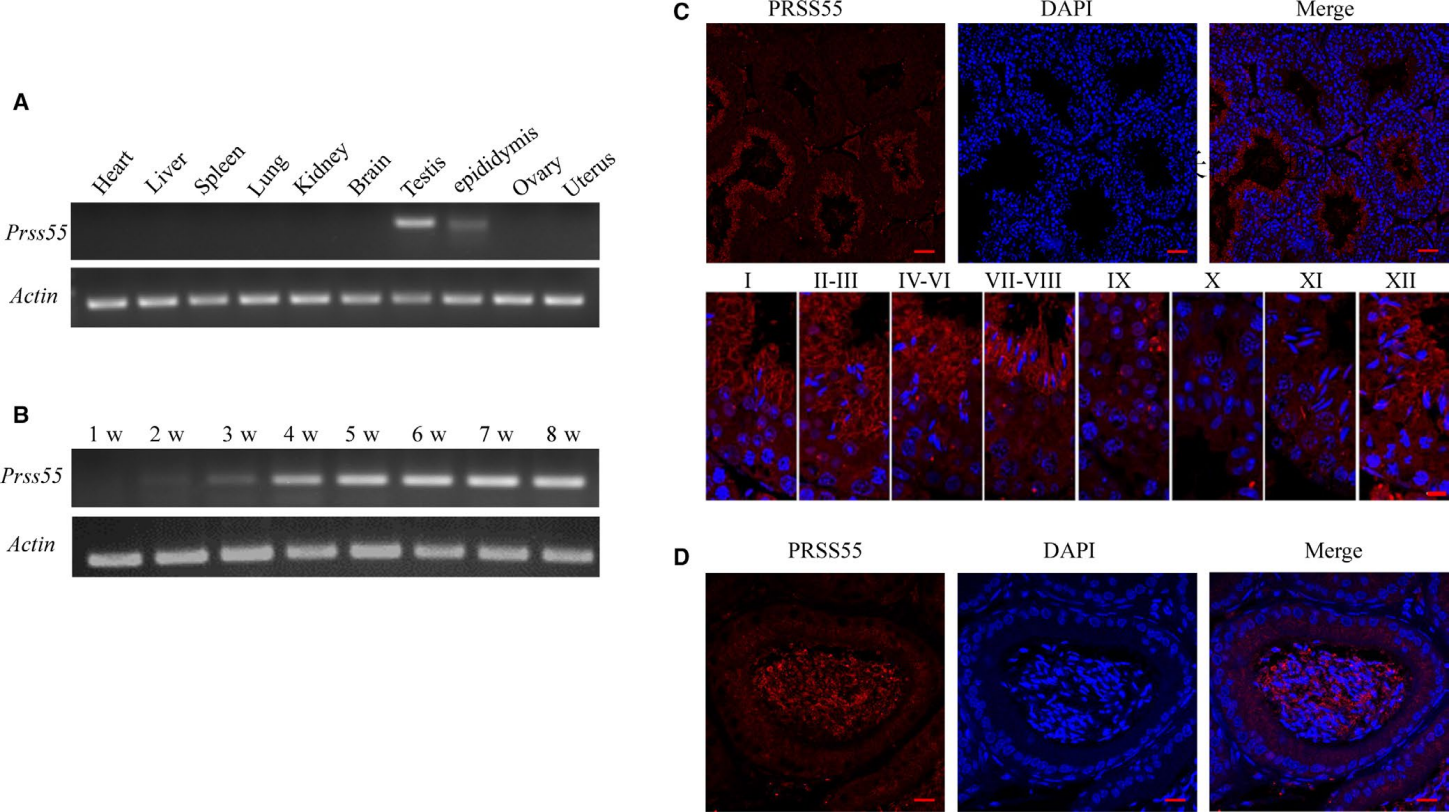
Expression and Location of the serine protease PRSS55 in mice. (A) Detection of the expression of *Prss55* mRNA in various tissues of mice, showing that it was only expressed in male reproductive organs. (B) The testicular *Prss55* mRNA expression profile was tested at the indicated time points after birth, demonstrating that its expression first appeared at approximately the second week. (C) Immunofluorescence localization of PRSS55 in the adult mouse testis showed it was restrictively expressed at steps 12‐16 of spermiogenesis. Each image exhibits a stage of the seminiferous epithelial cycle, denoted by Roman numerals at the top of each image. (D) Immunofluorescence localization of the PRSS55 protein in adult mouse epididymis, showing its expression in sperm but not in epithelial cells

The distribution of *Prss55* mRNA in various tissues of mice was detected using RT‐PCR. The cDNA samples were prepared from mouse tissues include heart, liver, spleen, lung, kidney, brain, testis, epididymis, ovary and uterus. As shown in Figure 1A, *Prss55* mRNA was only expressed in male reproductive organs, with prominent expression in the testis, but weak expression in the epididymis. Testis maturation is age‐dependent, and the first wave of spermatogenesis takes place within 35 postnatal days. To determine the temporal and spatial expression of *Prss55* mRNA in testis, we examined the *Prss55* mRNA abundance in testis of mice at 1, 2, 3, 4, 5, 6, 7 and 8 weeks old using RT‐PCR. The results showed that *Prss55* mRNA first appeared at the second week, and sharply increased at the third week, just before the initiation of spermatid elongation. *Prss55* expression then increased gradually until the adult stage (Figure 1B).

Immunofluorescence analysis was carried out to identify the expression and location of PRSS55 in adult mouse testis and epididymis. The results revealed that PRSS55 was restrictively expressed in the elongated spermatids at steps 12‐16 of spermiogenesis in the testis (Figure 1C). In the epididymis, PRSS55 was expressed in sperm rather than in epithelial cells (Figure 1D). Such a specific expression pattern indicated the potential physiological role of PRSS55 during spermatogenesis and sperm function in mice.

### 3.2 Generation of Prss55 knockout mice

**Figure 2:**
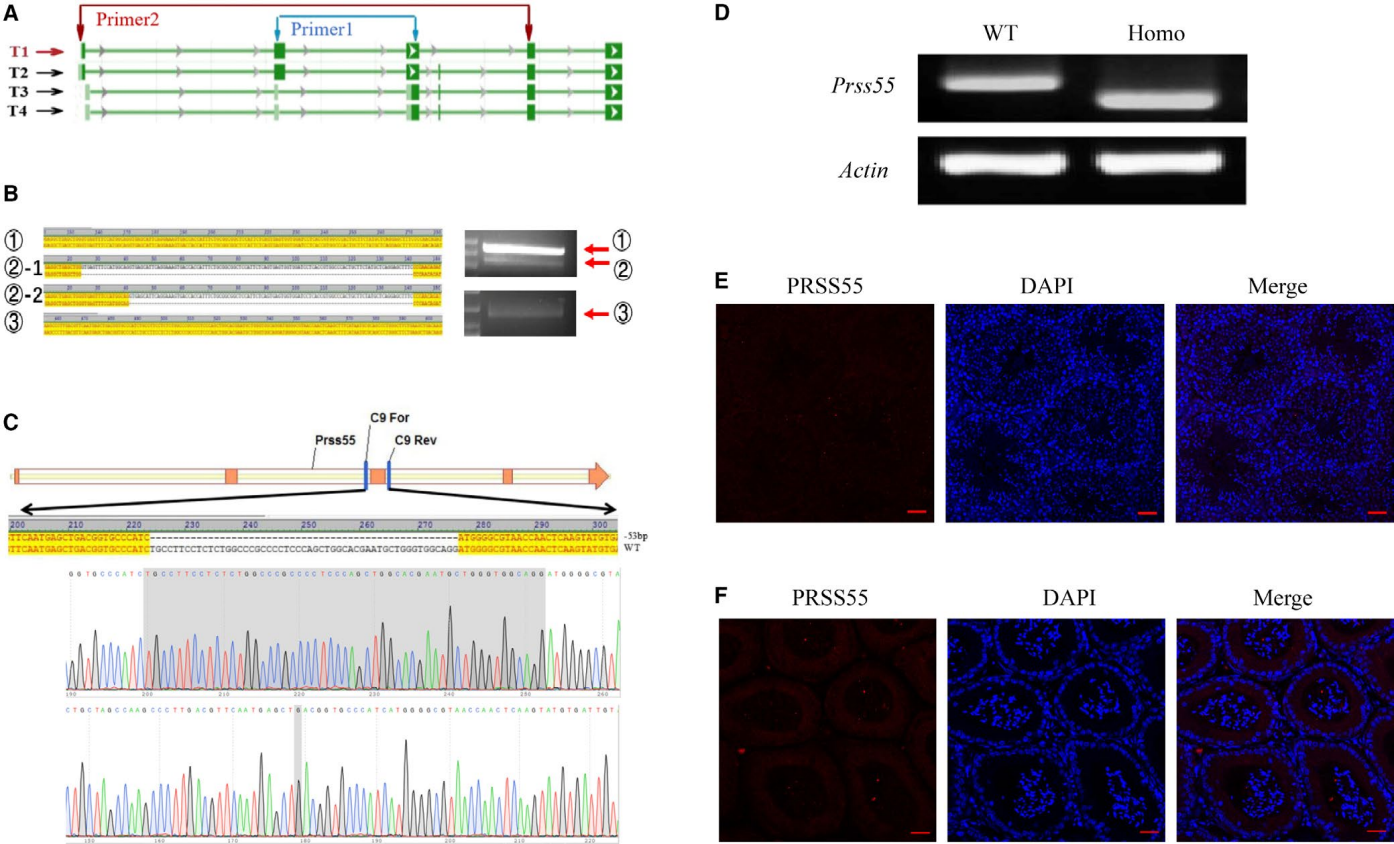
Detection of *Prss55* transcripts in mice testes and generation of *Prss55* gene knock out mice. (A) Schematic diagram of the possible pre transcriptional levels of *Prss55*. T1 is the known and confirmed transcript, and T2‐T4 are the predicted transcripts (NCBI). (B) We designed specific primer 1, which was located in exons 2 and 3, to detect all the transcripts of *Prss55*, primer 2 was designed to detect the T2 transcript, and PCR products were sequenced. (C) Schematic strategies for the generation of *Prss55* ^−/−^ (Homo) mice using CRISPR/Cas9 technology. 53 bp of *Prss55* were deleted from Exon 3. (D) The genotype of each Homo mouse was confirmed using PCR. (E) PRSS55 expression in the testes and epididymis of Homo mice was evaluated using immunofluorescence analysis, showing no positive signal in the elongating spermatids on one side of the tubule lumen. (F) PRSS55 expression in the epididymis of Homo mice was evaluated through immunofluorescence analysis, showing no positive signal in sperm. ① Corresponding to transcripts T1 and T2, ②‐1 and ②‐2 corresponding to transcripts T3 and T4, respectively, and ③ corresponding to transcript T2

By searching the NCBI database, we found that *Prss55* not only had a known and confirmed transcript, but also had several predicted transcripts. The proteins translated from these different transcripts were inconsistent in sequence, but all had a trypsin‐like serine protease domain. We confirmed that *Prss55* encoded all these different transcripts in mouse testes (Figure 2A,B).

Gene editing using CRISPR/Cas9 technology combined with microinjection technology was used to construct *Prss55* KO mice. We targeted the 3rd exon, which is common to all predicted transcripts, to design the sgRNA for zygote injection, which could achieve knockout in mice by targeting all transcripts of *Prss55* (Figure 2C). After the extraction of DNA, PCR amplification was performed using specific primers (Figure 2D). The results showed that a 53‐bp deletion from Exon 3 of *Prss55* could be detected. We then confirmed the absence of the PRSS55 protein in testis and epididymis of *Prss55* ^−/−^ mice using immunofluorescence assays (Figure 2E,F).

### 3.3 PRSS55 deficiency impaired sperm motility, thus affected fertility

**Figure 3:**
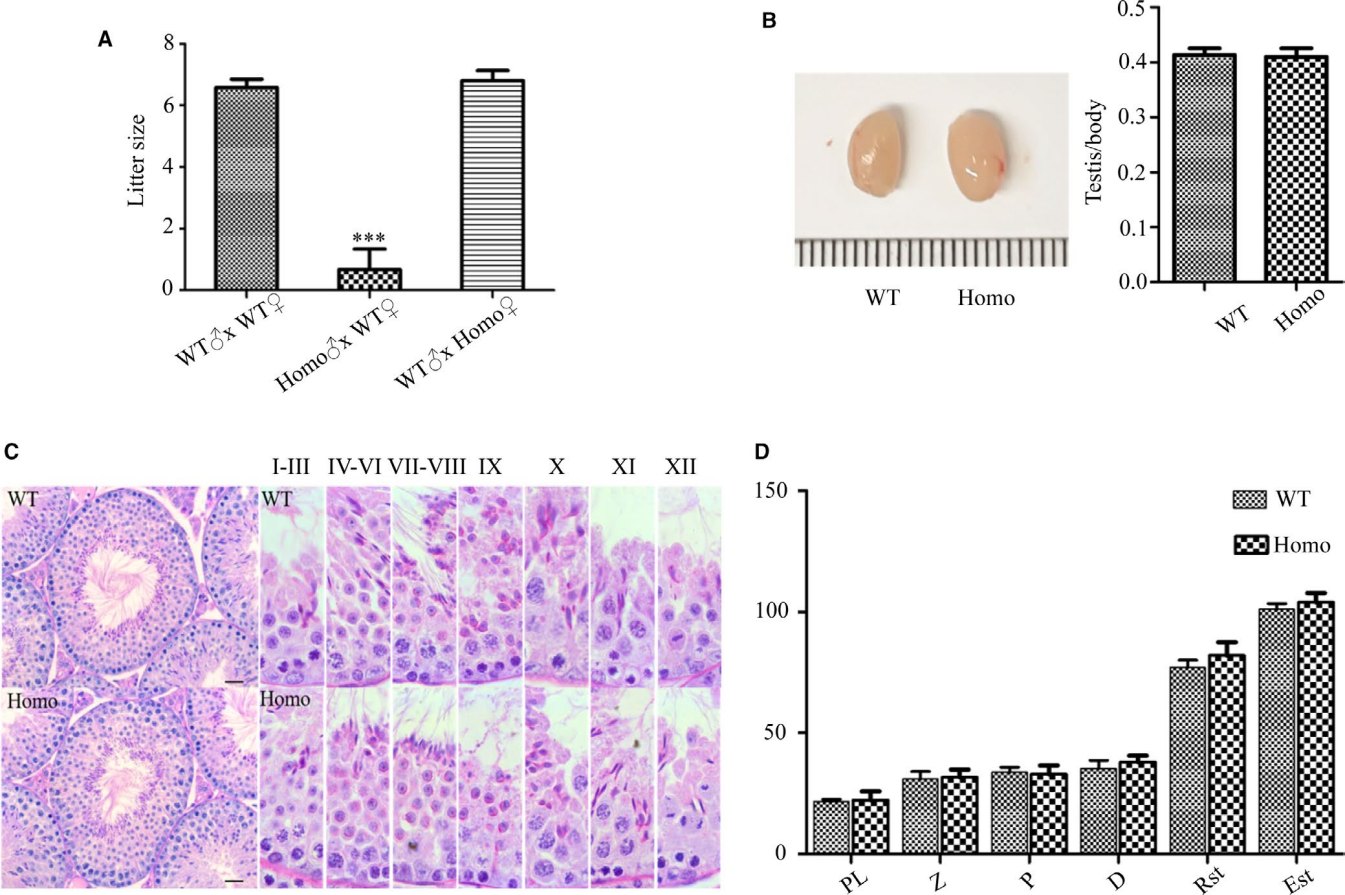
Evaluation of fertility and testicular structure. (A) Each male was mated with two females. Tests were performed for litter size on wild‐type (WT) and gene knock out mice, showing the serious damage of fertility in *Prss55* ^−/−^ (Homo) males. Data are presented as mean ± SD (n = 6). ***P < .001. (B) Morphology and size of WT and Homo mice testes showing no significant difference and the testis/body weight ratio for WT and Homo mice showing no significant difference (n = 3). (C) Haematoxylin and eosin (H&E)‐stained sections of testes from WT and Homo male mice. All the images showed normal spermatogenesis (n = 3). (D) The number of spermatogenic cells in the spermatogenic tubules of stages Ⅶ, Ⅷ and XI were counted and showed no significant difference between the WT and Homo mice (n = 3). PL: preleptotene, Z: zygotene, P: pachytene, D: diplotene, Rst: round spermatids, Est: elongated spermatids. Scale Bars, 20 μm

Mating tests were performed, and continuous monitoring for 6 months was used to analyse mouse fertility. We found that female *Prss55* ^−/−^ mice were fertile and had litter sizes comparable to WT mice. In contrast, *Prss55* ^−/−^ males exhibited a serious decline in fertility, only producing one pup during the monitoring period (Figure 3A) (*P* < .001). To determine the cause of the deficiency in fertility, we examined the male reproductive system.

**Figure 4:**
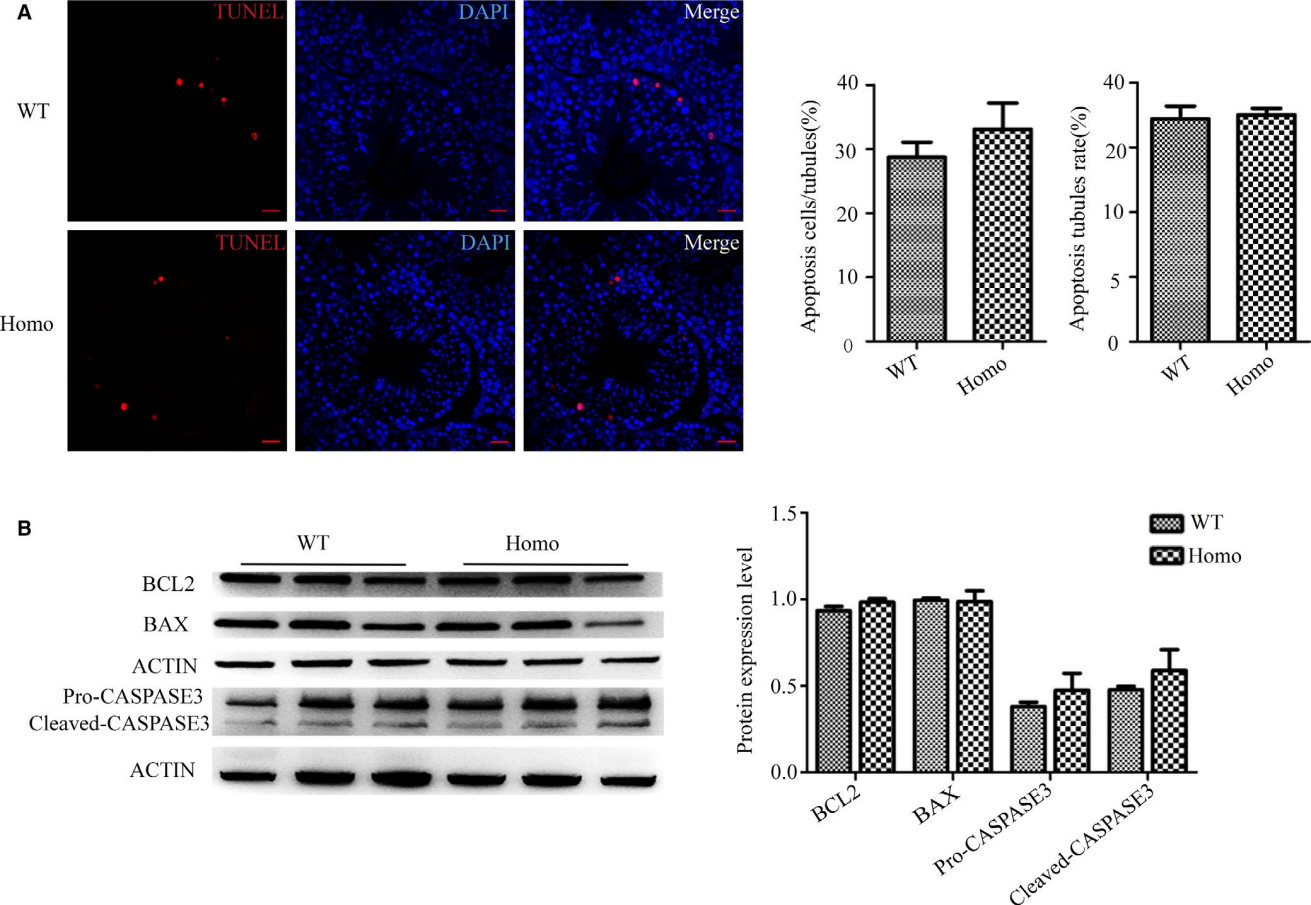
Apoptosis detection in the testis. (A) Terminal deoxynulceotidyl transferase nick‐end‐labelling (TUNEL) staining (red) in testes and the column diagram of statistics showing no significant difference (n = 3). Scale bars, 50 µm. (B) Apoptosis‐related molecules were detected in testis samples using Western blotting, the grey intensity analysis showed that their expression levels were not significantly different between the wild‐type (WT) and *Prss55* ^−/−^ (Homo) mice (n = 3)

The testis weight and size in *Prss55* ^−/−^ mice did not differ significantly from those of WT mice (Figure 3B). H&E staining of testis sections revealed that the arrangement and morphology of spermatogenic cells were similar between *Prss55* ^−/−^ and WT mice, showing no obvious abnormality (Figure 3C). The number of spermatogenic cells was counted under a light microscope, demonstrating similar results in *Prss55* ^−/−^ and WT mice (Figure 3D). In addition, apoptotic signals in the *Prss55* ^−/−^ mouse testes were not increased significantly compared with those in the WT controls (Figure 4A). BAX and BCL2 are the most important pair of proteins in apoptosis, which have opposite functions. BAX plays a pro‐apoptotic role, whereas BCL2 plays an anti‐apoptotic role. A series of regulations of the apoptotic pathway eventually activate caspase 3, which then promotes apoptosis. We found that the expression levels of these apoptosis‐related proteins in the KO mice were not significantly different from those in the control mice (Figure 4B). These results suggested that the absence of PRSS55 had no significant effect on apoptosis in the testis.

**Figure 5:**
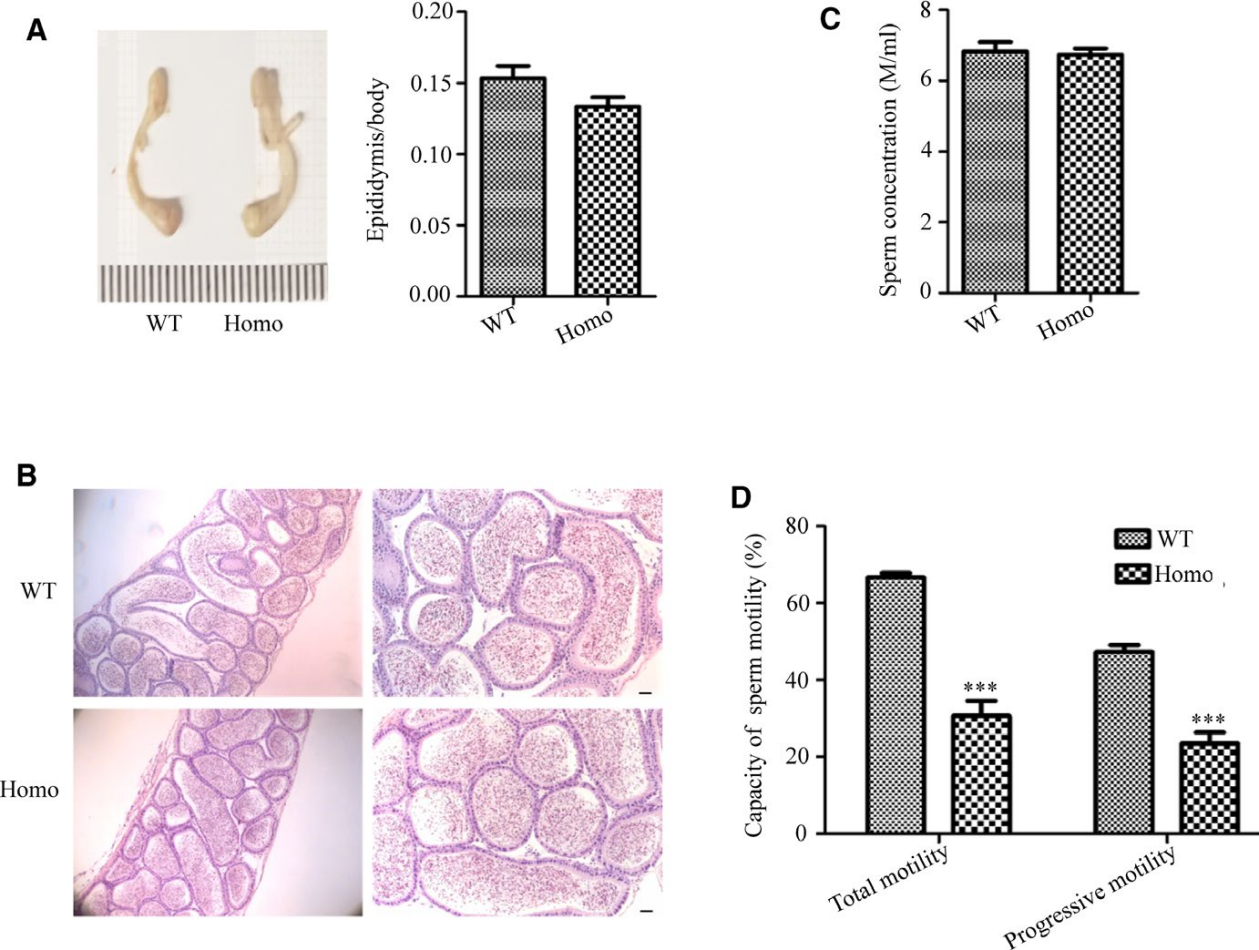
Evaluation of epididymis structure and sperm parameters. (A) The morphology and size of the epididymis, as well as the epididymis/body weight ratio, showed no significant difference between the wild‐type (WT) and *Prss55* ^−/−^ (Homo) mice (n = 3). (B) Observing the haematoxylin and eosin (H&E)‐stained sections showed similar histological structures of the epididymis in WT and Homo mice, with no significant abnormalities. (C) Sperm count from the cauda epididymis of WT and Homo mice showed no significant differences (n = 6). (D) Percentage of motile and progressively motile sperm from Homo mice decreased significantly (n = 6). ***P < .001

The morphology and histological structure of the epididymis also showed no obvious abnormalities in *Prss55* ^−/−^ mice compared with those in WT mice (Figure 5A,B). Further assessment of the indices of the epididymis sperm using CASA analysis showed that the sperm motility of *Prss55* ^−/−^ mice was significantly lower than that of the controls (Figure 5D) (*P* < .001), although there was no significant difference in sperm number between the two groups (Figure 5C).

### 3.4 Abnormal morphology, structure and ATP synthesis in Prss55^−/−^ mouse sperm

**Figure 6:**
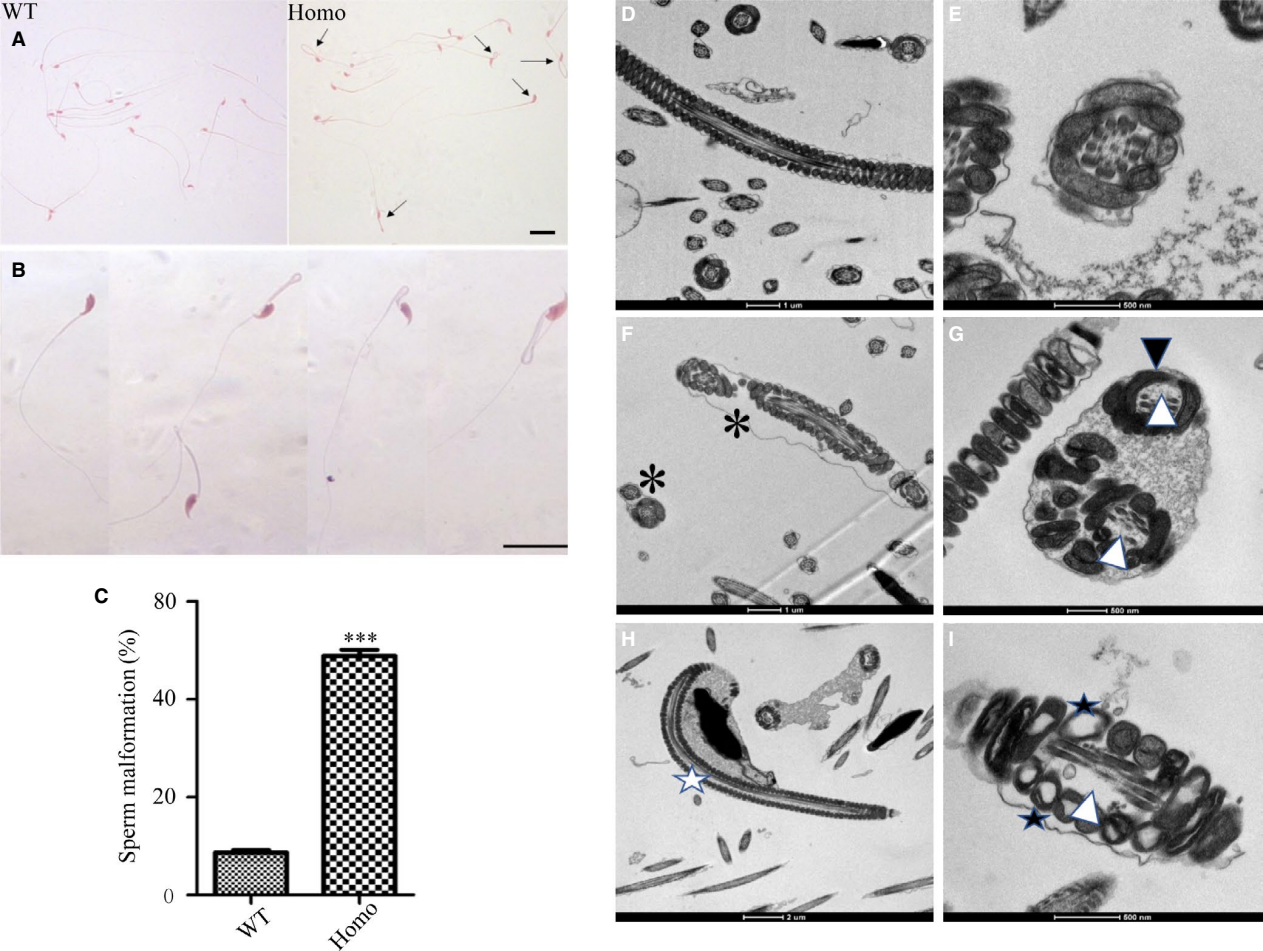
Morphological and ultrastructural observation of sperm. (A) The sperm morphology of the two groups of mice is shown using light microscopy at low magnification (the abnormal sperm is indicated by arrows). (B) Morphological abnormality of sperm in *Prss55* ^−/−^ (Homo) mice revealed at high magnification by light microscopy. Bar = 10 μm. (C) Statistical analysis showing that the sperm malformation rate in *Prss55* ^−/−^ male mice was significantly increased compared with that in wild‐type (WT) mice (n = 5). (D‐E) The normal structure of WT mice sperm tail was observed under a transmission electron microscope (D: longitudinal section; E: transverse section). (F‐I) The abnormal structure of *Prss55* ^−/−^ mice sperm under the transmission electron microscope (*: More than one set of flagellum structures contained in the same cross section; △: Absence of microtubules; ☆: Sperm head bending towards the tail; ▲: Abnormal peripheral dense fibres; ★: Lack of or blurred mitochondrial cristae).***P < .001

**Figure 7:**
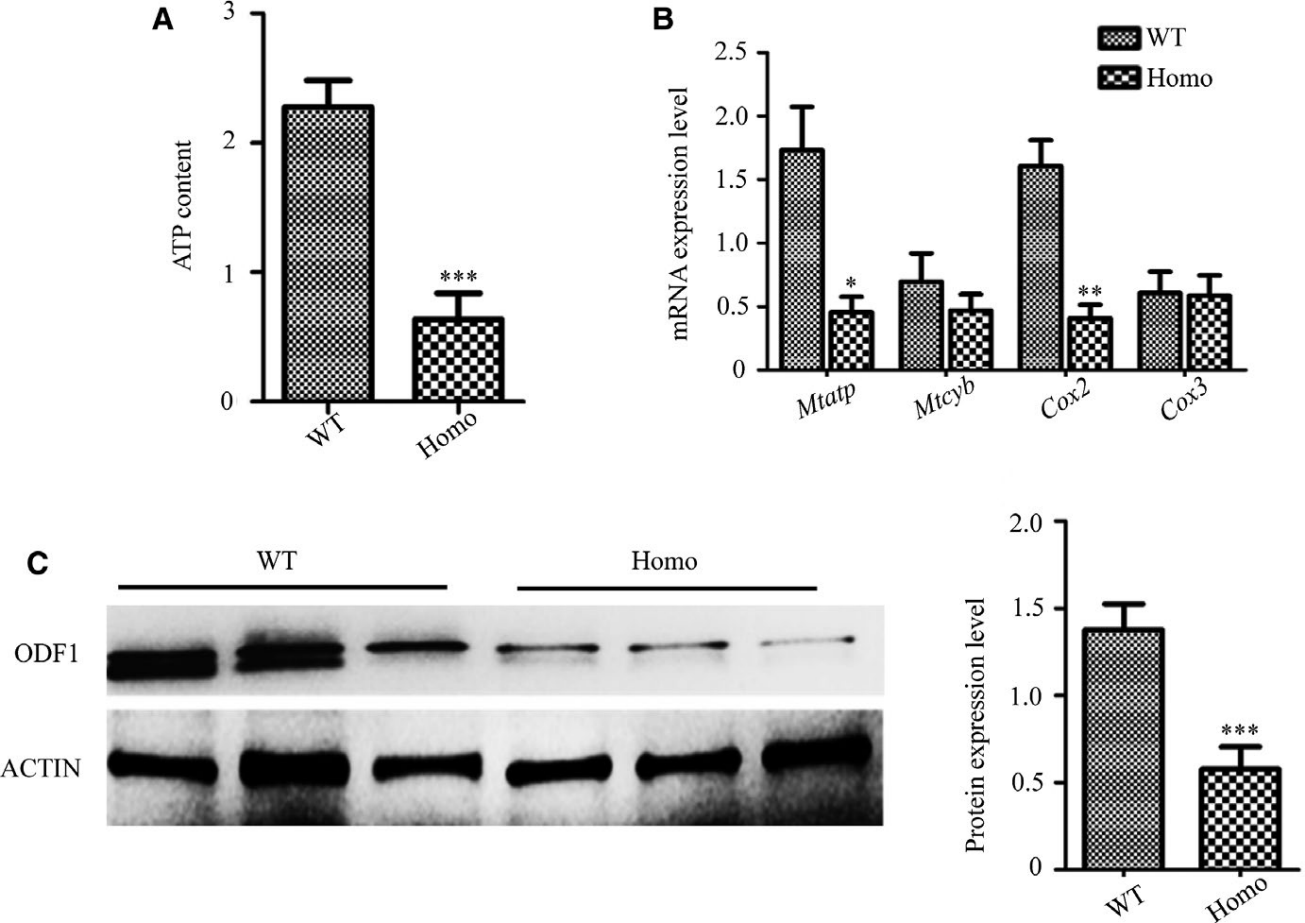
Molecular assessments of abnormal sperm structure and motility. (A) Detection of sperm ATP content showed a significant decrease in *Prss55* knockout mice (Homo) compared with that in wild‐type (WT) mice (n = 3). (B) The mRNA expression levels of key markers in the mitochondrial electron transfer chain in Homo sperm were obviously lower than those in the WT mice (n = 3). (C) Detection of ODF1 in sperm samples from WT and Homo mice using Western blotting; the grey intensity analysis shows an obvious decrease in Homo mice (n = 3). *P < .05, **P < .01, ***P < .001

To determine the reason for the decreased sperm motility in the *Prss55* ^−/−^ male mice, we first compared the morphology of sperm from the *Prss55* ^−/−^ and WT groups under a light microscope. The results showed that the sperm malformation rate in the *Prss55* ^−/−^ mice was significantly increased, mainly manifested as different degrees of folding and curling of the tail, and the sperm head was completely bent backward and closely fitted to the tail (Figure 6A‐C) (*P* < .001). We then compared the ultrastructure of the sperm from the two groups using transmission electron microscopy. Compared with WT mice (Figure 6D,E), markedly increased structural abnormality of sperm tails was observed in *Prss55* ^−/−^ mice, including more than one set of microtubules, peripheral dense fibres, and mitochondria in the same cross section of the sperm tail (Figure 6F), microtubule partial deletion (Figure 6G,I), inner mitochondrial membrane (IMM) cristae blur or lack (Figure 6I), and decreased peripheral dense fibres (Figure 6G). The incomplete peripheral dense fibres were also confirmed at the molecular level, in that outer dense fibre of sperm tails 1 (ODF1) levels in *Prss55^−/−^* mice sperm were significantly decreased compared with that in WT sperm (Figure 7C) (*P* < .001). In addition, ultrastructural analyses revealed that *Prss55* ^−/−^ mice sperm head was bent towards the tail and was tightly wrapped in a membrane (Figure 6H). These results were consistent with the morphological abnormality of sperm observed under the light microscope (Figure 6A,B).

To determine whether the abnormal mitochondria affect ATP generation and thus decrease sperm motility, we further detected the ATP content in the *Prss55* ^−/−^ and WT mice. The results showed that the ATP content of the *Prss55* ^−/−^ mice sperm was lower than that in the WT mice (Figure 7A) (*P* < .001). Considering that ATP is mainly produced by oxidative phosphorylation and the mitochondrial electron transfer chain is made up of four complexes, we thus detected several key markers of these complexes. The results showed that the mRNA levels of *Cox2* and *Mtatp* in *Prss55^−/−^* mice sperm were reduced compared those in the control (Figure 7B) (*P < .05, **P < .01).

### 3.5 Identification of potential target substrate of PRSS55

**Figure 8:**
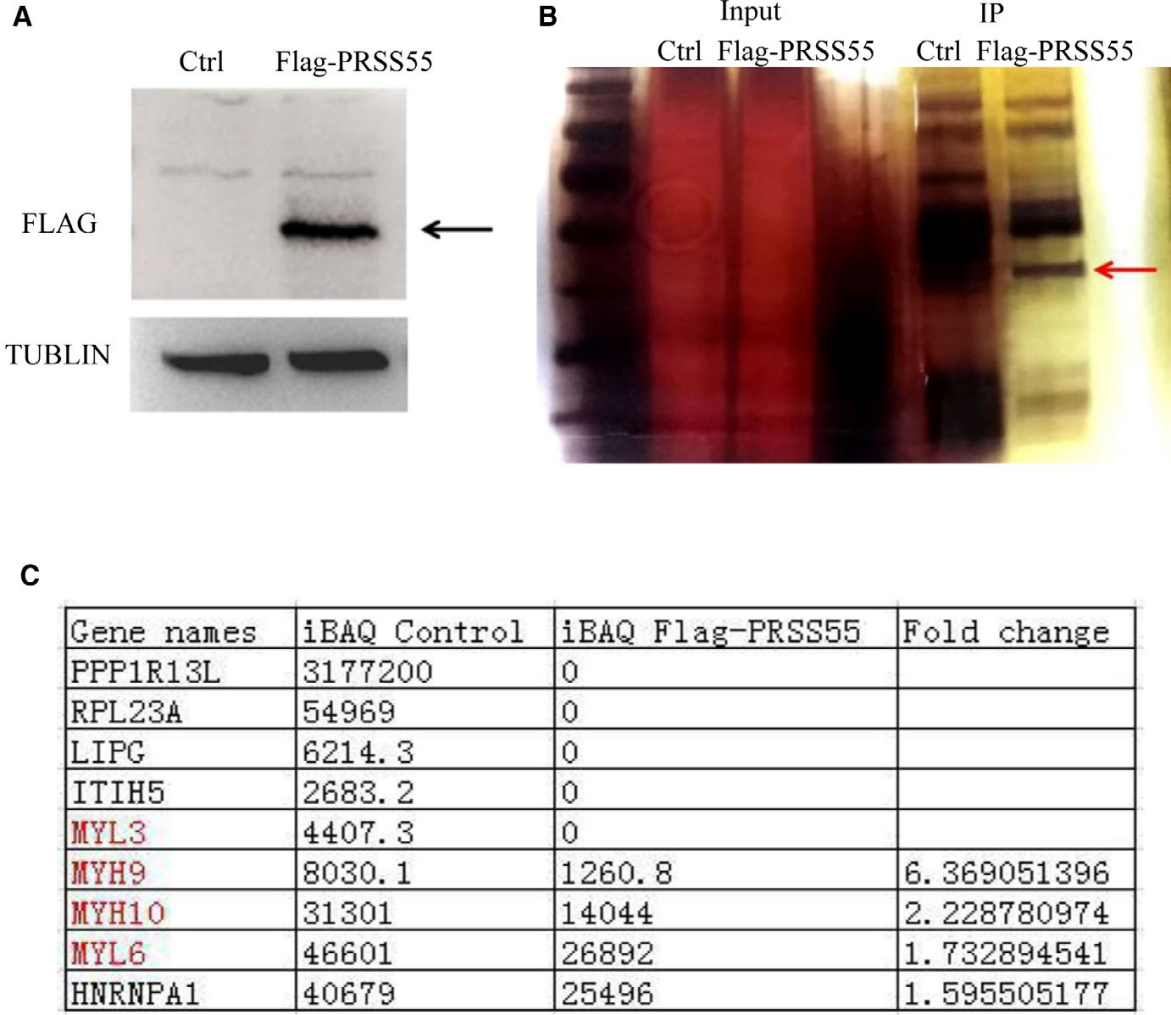
Identification of potential target substrates of PRSS55. (A) PRSS55 protein (black arrow) was successfully expressed in HEK293T cells. (B) PRSS55 and proteins that might interact with PRSS55 (red arrow) were eluted. (C) The proteins in the immunoprecipitation eluent were identified using mass spectrometry. Five proteins were found to be absent and four proteins showed decreased levels (>1.5 fold) in the experimental group compared with those in the control group

A PRSS55 overexpression plasmid expressing PRSS55 with a Flag label was transfected into HEK293T cells. After transfection of the PRSS55 expression plasmid, the cellular proteins were extracted, and anti‐Flag antibodies were used to detect the transfection efficiency via Western blotting. The results showed that PRSS55 was successfully expressed in HEK293T cells (Figure 8A). An immunoprecipitation experiment was carried out with proteins extracted from HEK293T cells transfected with the PRSS55‐Flag plasmid. The result showed that PRSS55 and proteins that might interact with PRSS55 were eluted (Figure 8B). The eluate obtained by immunoprecipitation was subjected to mass spectrometry and several proteins were considered to be the potential substrates of PRSS55, which showed significantly decreased levels (>1.5 fold) or were absent in the experimental group, including several type II muscle myosin family members (MYL3, MYL6, MYH9 and MYH10) (Figure 8C). We further examined the expression of these four molecules in the testis and sperm, and found that *Myl3*, *Myl6*, *Myh9* and *Myh10* were expressed in the testis (Figure S1), but only *Myl6*, *Myh9* and *Myh10* were expressed in sperm (Figure S2).

## 4 DISCUSSION

Based on the characteristics of proteases, they have been confirmed as key regulators of protein function and participate in various physiological functions.^3^, ^5^ The male reproductive system contains abundant serine proteases, which implies the important role of proteases in spermatogenesis and sperm function. Several members of the serine protease family have been reported to play an important role in male reproduction, participating in spermatogenesis or playing a role in different aspects of sperm function.^4^, ^5^, ^6^, ^7^, ^8^, ^9^, ^10^, ^11^, ^12^, ^13^, ^14^, ^15^, ^16^ However, the roles of other members remain unknown, or research has shown inconclusive results.

This study focused on the serine protease PRSS55. Similar to previous reports,^14^, ^16^ our results showed that PRSS55 was specifically expressed in testis spermatids and epididymis sperm. This specific expression pattern suggested an important role of PRSS55 in spermiogenesis and sperm function. Three teams had investigated the role of PRSS55 in male reproduction previously, with inconsistent. Khan et al found that the deletion of PRSS55 did not affect the fertility of male mice.^15^ However, both Shang's team and Kobayashi's team believed that PRSS55 was essential for male reproduction, and they found that deficiency of PRSS55 affected specific sperm functions (including sperm‐uterotubal junction (UTJ) migration and sperm‐ZP binding), which impaired fertility, although their test results in some experimental indicators were different.^14^, ^16^ There are four transcripts of *Prss55* in the testis, and failure to knock out all of them might lead to incomplete or unstable phenotypes. In this study, we constructed a gene knockout mouse model that targeted all the transcripts of *Prss55*, which theoretically, could reveal the role of PRSS55 more comprehensively. Through a series of experiments, we attempted to determine whether PRSS55 is necessary for male fertility and whether it is involved in other aspects of spermatogenesis or sperm function.

The *Prss55* ^−/−^ male mice exhibited a serious decrease in fertility. Analyses of the structure of reproductive organs, the number of germ cells, as well as the basic sperm indices in these mice, demonstrated that the decline in male fertility was not caused by a defect of sperm quantity, but by impaired sperm quality, manifesting as a significant decline in motility.

The motility of sperm depends on its differentiated normal tail structure (such as microtubules, peripheral dense fibres and mitochondria) and energy metabolism. The "9 + 2" microtubule complex constitutes the axoneme, which runs through the neck of sperm to the whole flagellum, and plays an important role in flagellum movement. The peripheral dense fibres contain abundant cysteine and disulphide bonds, which are very important for the stability and elastic retraction of sperm flagellum. The mitochondria wrap around the peripheral dense fibres in a spiral way to form a mitochondrial sheath, and ATP is produced for sperm movement by mitochondrial metabolism.^19^

Thus, the sperm tail is an important structure to ensure normal sperm movement. In this study, we found that the mice lacking PRSS55 showed an abnormal extension and damaged structure of sperm tails. In these mice, sperm tails exhibited different degrees of folding and curling, and movement‐related structures in the sperm tails all exhibited abnormalities, including deficiency of “9 + 2” microtubules, damage to peripheral dense fibres, and defects of mitochondrial cristae. We confirmed the above abnormal phenotypes from different aspects including morphological observation, structural assessment and molecular detection (such as the level of ODF1). Furthermore, abnormal energy metabolism in the *Prss55* KO mice was also confirmed: their sperm ATP content was significantly lower than that of the WT mice. The energy for sperm motility comes from ATP produced by anaerobic glycolysis and aerobic oxidation, among which aerobic oxidation produces much more ATP via the oxidative respiratory chain in inner mitochondrial membrane cristae.^20^ We found that the expression levels of COX2 and MTATP, the key molecules in the oxidative respiratory chain, decreased significantly in *Prss55* ^−/−^ mice sperm.

The above results explained the decrease of sperm motility in *Prss55* ^−/−^ mice. In Shang et al's and Kobayashi et al's studies, there was no abnormality in testicular structure and sperm number of *Prss55* KO mice, which was the same as our results. However, they did not find defects in sperm morphology and structure, or a decrease in sperm motility. They demonstrated that sperm migration through the UTJ was impaired, and explained this phenomenon by the reduced level of mature ADAM metallopeptidase domain 3 (ADAM3) in sperm (ADAM3 is thought to play a pivotal role in sperm migration through the female reproductive tract^21^). We also confirmed the reduced expression of ADAM3 in the sperm of *Prss55* ^−/−^ mice (Figure S3). We hypothesized that the declined motility of sperm itself is also an important reason for the defect in its UTJ migration. Studies have demonstrated that sperm movement ability plays an important role in its passage through the vagina, uterus, UTJ and oviduct.^22^ Thus, the defect in sperm motility provides a further explanation for the subfertility phenotype of *Prss55* ^−/−^ mice.

Our results indicated that PRSS55 might participate in the differentiation of spermatids and directly affect sperm function, which was consistent with the specific expression characteristics of PRSS55 in testicular spermatids and epididymal spermatozoa. Kobayashi et al proposed that PRSS55 is expressed in testicular germ cells and retained in epididymis sperm, but might play a key role in epididymis sperm rather than testicular germ cells.^14^ We think that PRSS55 functions both in the testicle and the epididymis. Compared with the results of Shang and Kobayashi groups, additional sperm phenotypes of *Prss55* ^−/−^ mice were found in the present study. We think this was because of the different gene knockout strategy. We knocked out all the transcripts of *Prss55* in testis, whereas the previous studies only knocked out two of them.

In summary, PRSS55 is involved in the structural differentiation and motility of sperm. As a protease, it should function by activating substrate proteins. This study attempted to identify the possible substrates of PRSS55. After immunoprecipitation and mass spectrometry analysis, we identified several type II muscle myosin family members (MYL3 MYL6 MYH9 and MYH10), which might be the precursor protein substrates of PRSS55. Myosin forms a dynein superfamily that binds to actin and generates power by hydrolysis of ATP. Myosin is divided into many categories, among which type II myosin includes myocyte myosin and non‐muscle myosin.^23^ Type II muscle myosin is expressed in all eukaryotic cells and is involved in many process requiring motivation and cytoskeleton translocation, such as cell migration, polarization, adhesion and maintenance of cell morphology, and signal transduction.^24^, ^25^, ^26^ We confirmed the expression of these four potential substrates in the testis and sperm at the mRNA level (*Myl3*, *Myl6*, *Myh9* and *Myh10* were expressed in testis but only *Myl6*, *Myh9* and *Myh10* were expressed in sperm), indicating that PRSS55 may have different functional substrates in testis and sperm. As a serine protease, PRSS55 might function by activating these II muscle myosins in the testis, which then participate in sperm structural differentiation and further affect its function, a defect in which could lead to male infertility.

This study used a more comprehensive strategy to construct *Prss55* KO mouse model to investigate the important role of PRSS55 in male fertility. Our results demonstrated that PRSS55 is essential for the structural differentiation and function maintenance of sperm. PRSS55 is likely to be a potential pathogenic factor in male astheno/teratozoospermia. More details of the mechanism of action of PRSS55 remain to be determined.

## CONFLICT OF INTEREST

The authors confirm that there are no conflicts of interest.

## AUTHOR CONTRIBUTIONS

**Feng Zhu:** Data curation (equal); Writing‐original draft (equal). **Wen Li:** Data curation (equal); Writing‐original draft (equal). **Xinli Zhou:** Data curation (supporting); Resources (supporting). **Xu Chen:** Formal analysis (supporting). **Meimei Zheng:** Formal analysis (supporting). **Yiqiang Cui:** Methodology (supporting). **Xiaofei Liu:** Methodology (supporting). **xuejiang guo:** Methodology (lead). **Hui Zhu:** Project administration (lead); Supervision (lead).

## Supporting information

Supplementary Material

## Data Availability

This is an open access article under the terms of the Creative Commons Attribution License, which permits use, distribution and reproduction in any medium, provided the original work is properly cited.
