# Supplementary material for: PRSS55 plays an important role in the structural differentiation and energy metabolism of sperm and is required for male fertility in mice
Source: J Cell Mol Med. 2021 Jan 8;25(4):2040–51. doi: 10.1111/jcmm.16116 (PMC7882947; doi:10.1111/jcmm.16116)
Supplement: Supplementary file 1 — Supplementary Material [file JCMM-25-2040-s001.doc]

**Supplemental Table 1. Expression of PRSS family members in different organs and tissues**

| Classification of tissues | Family members |
| --- | --- |
| Brain | PRSS9/18, PRSS12; |
| Endocrine tissues | PRSS15, PRSS36, PA; |
| Bone marrow & immune system | PRSS8, PRSS25, PRSS56, PRSS57; |
| Pancreas | PRSS1, PRSS2, PRSS3, PRSS4, PRSS16; |
| Gastrointestinal tract | PRSS7, PRSS14, PRSS19, PRSS20, PRSS22, PRSS26, PRSS27, PRSS31; |
| Male tissues | PRSS10, PRSS17, PRSS21, PRSS24, PRSS37, PRSS38, PRSS41, PRSS42, PRSS43, PRSS44, PRSS45, PRSS46, PRSS48, PRSS50, PRSS54, PRSS55, PRSS58, ACR, PCSK4, PA, HTRA2; |
|
| Female tissues | PRSS5, PRSS11, PRSS23, PRSS33, PRSS35; |
| skin | PRSS6, PRSS53; |
| PS: Only expressed in humans: PRSS4, PRSS10, PRSS26;  Only expressed in mice: PRSS28, PRSS29, PRSS30, PRSS32, PRSS34, PRSS39,  PRSS40, PRSS49,PRSS52;  The others are expressed in both humans and mice. | |
|
|

**Supplemental Table 2. Primer sequences of genes used in the present study**

| Gene name | Primer sequences (5′-3′) |
| --- | --- |
| Primer1 | CCAGGATCATAGAAGGGCAGGAG  GGCACCGTCAGCTCATTGAAC |
| Primer2 | GGGAGTCTGAGCTGCCAGTTGA  CGTTGACATAGATTCCTTGTCAGCTTC |
| Prss55 | GGAAGCAAATGTTGAGTGTGGT  TTCCTGAATGCTCACCTGCC |
| Cox2 | AATTGCTCTCCCCTCTCTACG  GGTGCCCTATGGTTTTAACG |
| Cox3 | AGGCCACCACACTCCTATTG  AGCAGCCTCCTAGATCATGTG |
| Mtcyb | ATTCCTTCATGTCGGACGAG  CTGTGGCTATGACTGCGAAC |
| Mtatp | CTCACTTGCCCACTTCCTTC  GTAAGCCGGACTGCTAATGC |
| β-actin | TGACCCAGATCATGTTTGAGACC  ATAGATGGGCACAGTGTGGG |
| Adam3 | CCGAGACCCAGATAGGCAGTGT  CCCGTGTCCTTGGCATTTGTCA |
| Myl3 | TCCAGCACATCTCCAAGAACA  AAGGCAAGCACAGGTAGGTAA |
| Myh9 | AACGCCAAGACGGTGAAGA  CCAGACAGCAGGTAGTAGAAGA |
| Myh10 | GAGGAGGAGCAGAGCAACAT  AGATGGTAGCCTTGAACTTGGA |
| Myl6 | GACTTCACCGAGGACCAGAC  CAGCACCTTCACATTCATCTCA |


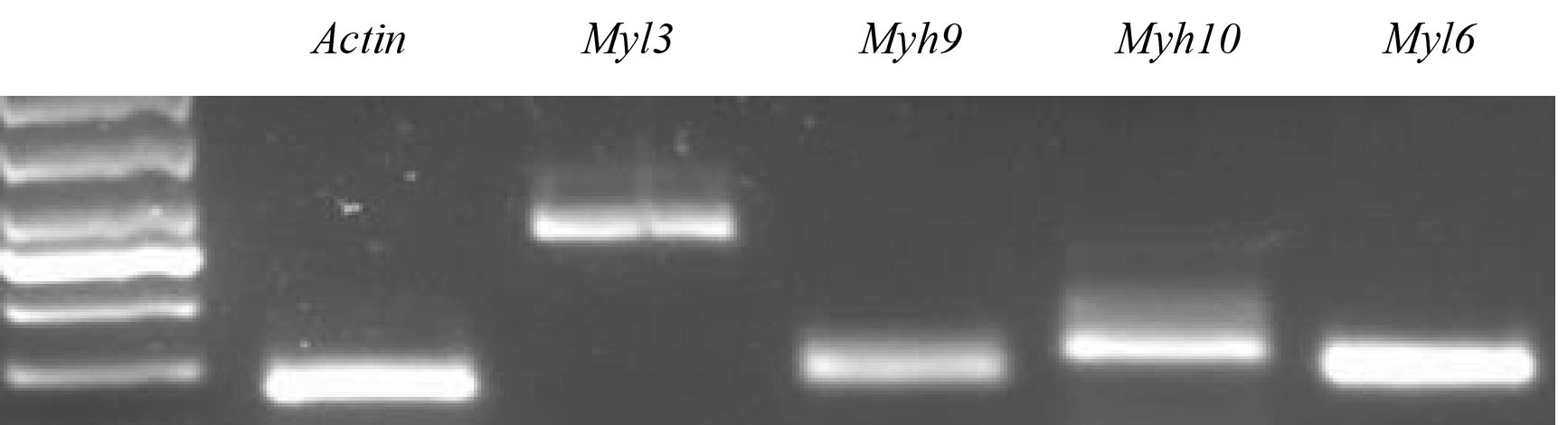


Supplemental Figure 1. Expression of *Myl3*, *Myh9*, *Myh10*, and *Myl6* in the testis of wild-type (WT) mice.

*Myl3*, *Myh9*, *Myh10*, and *Myl6* were all expressed in wild-type mouse testis.


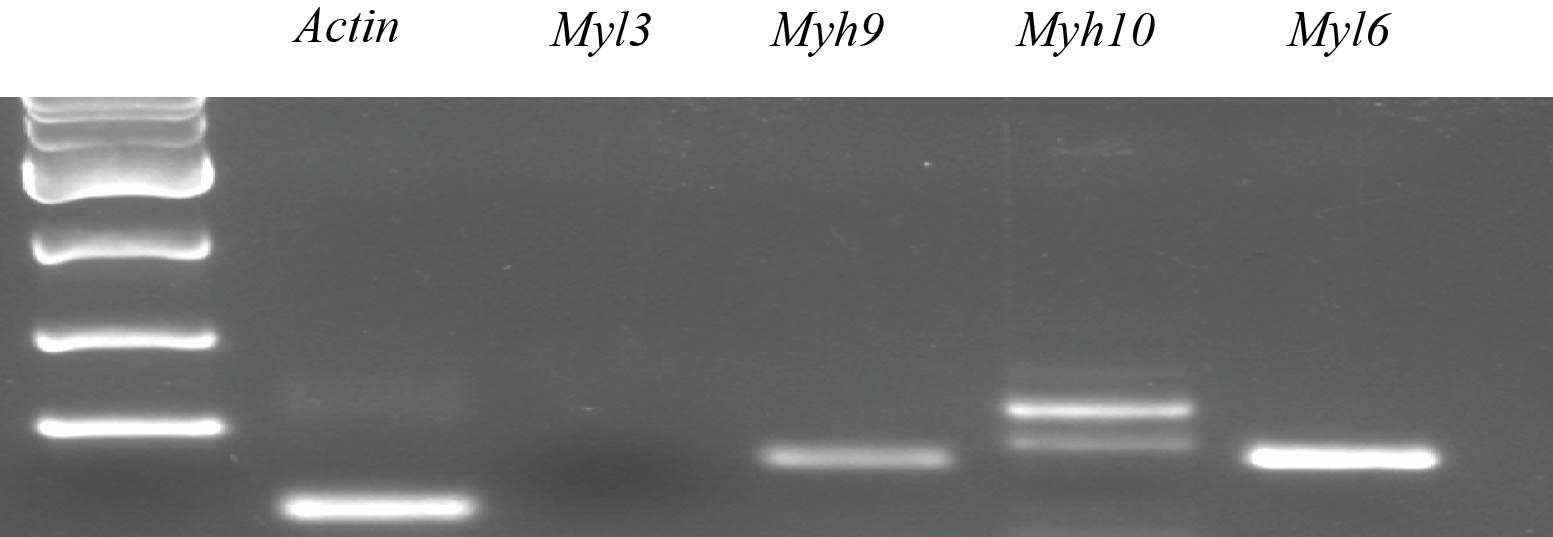


Supplemental Figure 2.Expression of *Myl3*, *Myh9*, *Myh10*, and *Myl6* in the sperm of wild-type (WT) mice.

Only *Myh9*, *Myh10*, and *Myl6* were expressed in wild-type mouse sperm.

**
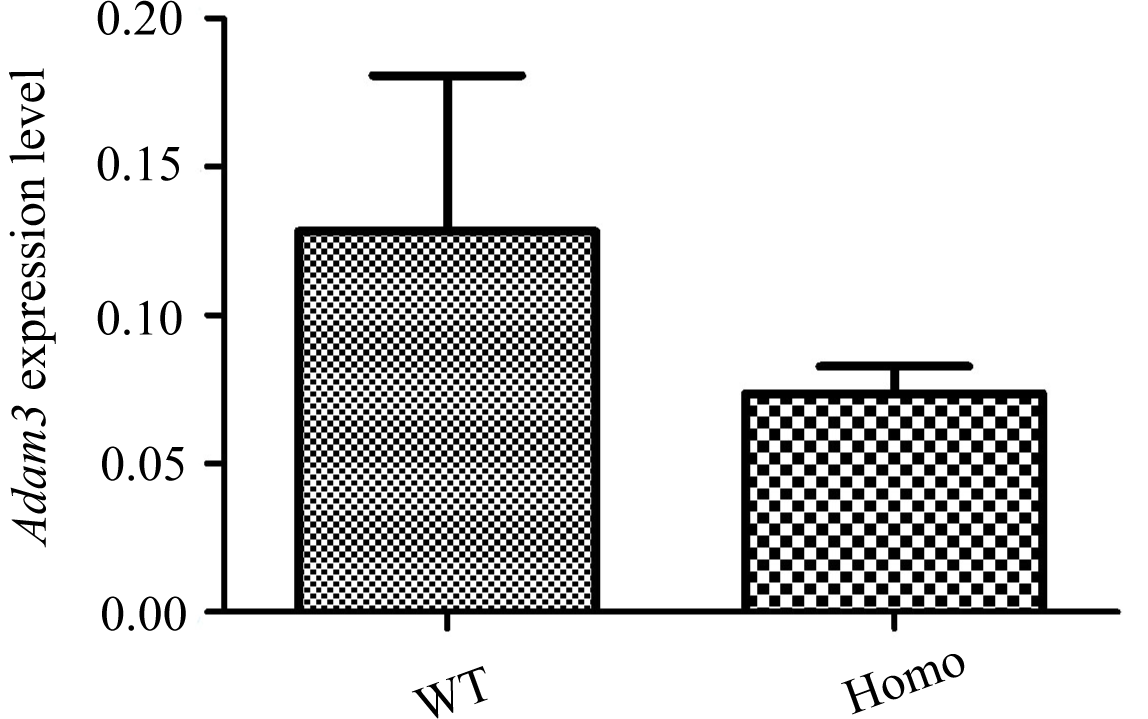
**

Supplemental Figure 3. Expression of *Adam3* in the sperm of wild-type (WT) and *Prss55* knockout mice.

The mRNA level of *Adam3* in *Prss55* knockout (Homo) mouse sperm was lower than that in WT mice (n = 3).
